# On the Alloying and Properties of Tetragonal Nb_5_Si_3_ in Nb-Silicide Based Alloys

**DOI:** 10.3390/ma11010069

**Published:** 2018-01-04

**Authors:** Panos Tsakiropoulos

**Affiliations:** Department of Materials Science and Engineering, University of Sheffield, Sheffield S1 3JD, UK; p.tsakiropoulos@sheffield.ac.uk

**Keywords:** silicides, intermetallics, alloying, hardness, creep

## Abstract

The alloying of Nb_5_Si_3_ modifies its properties. Actual compositions of (Nb,TM)_5_X_3_ silicides in developmental alloys, where X = Al + B + Ge + Si + Sn and TM is a transition and/or refractory metal, were used to calculate the composition weighted differences in electronegativity (Δχ) and an average valence electron concentration (VEC) and the solubility range of X to study the alloying and properties of the silicide. The calculations gave 4.11 < VEC < 4.45, 0.103 < Δχ < 0.415 and 33.6 < X < 41.6 at.%. In the silicide in Nb-24Ti-18Si-5Al-5Cr alloys with single addition of 5 at.% B, Ge, Hf, Mo, Sn and Ta, the solubility range of X decreased compared with the unalloyed Nb_5_Si_3_ or exceeded 40.5 at.% when B was with Hf or Mo or Sn and the Δχ decreased with increasing X. The Ge concentration increased with increasing Ti and the Hf concentration increased and decreased with increasing Ti or Nb respectively. The B and Sn concentrations respectively decreased and increased with increasing Ti and also depended on other additions in the silicide. The concentration of Sn was related to VEC and the concentrations of B and Ge were related to Δχ. The alloying of Nb_5_Si_3_ was demonstrated in Δχ versus VEC maps. Effects of alloying on the coefficient of thermal expansion (CTE) anisotropy, Young’s modulus, hardness and creep data were discussed. Compared with the hardness of binary Nb_5_Si_3_ (1360 HV), the hardness increased in silicides with Ge and dropped below 1360 HV when Al, B and Sn were present without Ge. The Al effect on hardness depended on other elements substituting Si. Sn reduced the hardness. Ti or Hf reduced the hardness more than Cr in Nb_5_Si_3_ without Ge. The (Nb,Hf)_5_(Si,Al)_3_ had the lowest hardness. VEC differentiated the effects of additions on the hardness of Nb_5_Si_3_ alloyed with Ge. Deterioration of the creep of alloyed Nb_5_Si_3_ was accompanied by decrease of VEC and increase or decrease of Δχ depending on alloying addition(s).

## 1. Introduction

Performance and environmental targets for future aero-engines could be met with changes in the propulsive and thermal efficiency of the engines and new materials that have capabilities beyond those of Ni-based superalloys. Industry has set the following property goals for new ultra-high temperature alloys with capabilities beyond those of Ni-based superalloys: the room temperature fracture toughness must be above 20 MPa(m)^1/2^, there must be less than 1% creep in 125 h at 1473 K and σ > 170 MPa (alloy density ρ = 7 g/cm^3^) and the oxidation life at 1588 K must be equal to that of second generation single crystal Ni-based superalloys at 1423 K, with a short term oxidation goal to have sufficient oxidation resistance in the uncoated condition to survive under typical engine conditions, which requires a loss of material less than 200 μm thickness in 10 h at 1643 K and a long term oxidation goal that requires a loss of material less than 25 μm thickness in 100 h at 1588 K [1].

Refractory metal intermetallic composites (RMICs) have the potential to offer a balance of properties required in critical applications in future aero-engines. RMICs can be in situ composites. Composite microstructures can be formed in situ with TM_5_Si_3_ (TM = transition and refractory metal) silicides. Interest in the TM_5_Si_3_ silicides for structural materials is justified because of their high temperature strength and creep properties, their high melting points, which are in excess of 2273 K and their solubility ranges [2], which make alloying with different elements possible. The 5-3 silicides of the transition metals of groups IV to VI have the D8_8_ (hP16, Mn_5_Si_3_ prototype), D8_l_ (tI32, Cr_5_B_3_ prototype), or D8_m_ (tI32, W_5_Si_3_ prototype) structure types. These three structures have different numbers of non-equivalent sites (three for D8_8_ and four for D8_l_ and D8_m_). The 5-3 silicides of the group IV elements (Ti, Zr, Hf) have the D8_8_ structure. Those of the group V elements Nb and Ta have the D8_m_ structure at high temperature and the D8_l_ structure at low temperature. Vanadium and the group VI elements Cr, Mo and W have the D8_m_ structure.

The motivation for pursuing in situ compositing is the poor toughness of the 5-3 silicide(s) at room temperature and the fact that with 5-3 silicides there are broad alloying opportunities that can lead to suppression of other silicide(s) and the formation of eutectics between 5-3 silicides and refractory metal solid solution(s) [3,4,5,6]. The properties of 5-3 silicides depend on alloying. For example, the coefficient of thermal expansion (CTE) of 5-3 silicides, including Nb_5_Si_3_, can be anisotropic and the CTE anisotropy is changed by alloying. Control of the CTE anisotropy of TM_5_Si_3_ silicides via alloying is important because CTE anisotropy is expected to have a negative effect on the processing of the alloys and a negative effect on the life of components, owing to the residual micro-stresses at grain boundaries at temperatures below the ductile to brittle transition temperature [7].

The ratio (CTE_c_)/(CTE_a_) of the coefficients of thermal expansion along the c and a axes of different 5-3 silicides is used to show their CTE anisotropy. This ratio is given in the Table 1 for different 5-3 silicides. The different experimental data for the CTE anisotropy of Ti_5_Si_3_, Mo_5_Si_3_, Zr_5_Si_3_ and αNb_5_Si_3_ could be attributed to the difficulties in making arc melted alloys with homogeneous microstructures [20]. Alloying the Ti_5_Si_3_ with Nb or Ta or Ge did not change the CTE anisotropy but additions of B, Cr, Hf, V and Zr changed it to about 2 [9,10]. Alloying with B had a strong effect on the CTE anisotropy of W_5_Si_3_ (T2 phase) which was reduced to about 1.1 [13]. The data in Table 1 shows that the alloying of Nb_5_Si_3_ with Ti also changed its CTE anisotropy and that these changes are not as dramatic as those for alloyed Ti_5_Si_3_. Contamination of the 5-3 silicides by interstitials also can change their CTE anisotropy. For example, in the case of Ti_5_Si_3_ contamination by C or N changed the anisotropy ratio to about 2 but contamination by O had no effect on the CTE anisotropy [9]. 

Nb-silicide based alloys, which are also known as Nb-silicide in situ composites, are RMICs that can surpass the fracture toughness and creep property goals and their oxidation can be close to the oxidation goal [1,3,21]. Reductions in rotor weight of more than 20% could be realized through the substitution of Nb-silicide based aerofoils for Ni-base superalloys aerofoils in present and advanced turbine designs [1,21]. The most important phases in the microstructure of Nb-silicide based alloys are the bcc Nb solid solution(s), Nb_ss_ and the Nb_5_Si_3_ silicide. The latter can be present as the tetragonal high temperature βNb_5_Si_3_, or the tetragonal low temperature αNb_5_Si_3_ [2], or as the hexagonal γNb_5_Si_3_ silicide. The γNb_5_Si_3_ is not desirable owing to its creep properties [1]. The Nb_ss_ can be rich in Ti [4] or free of Si [22]. Other phases also can be stable in Nb-silicide based alloys, for example the C14-NbCr_2_ Laves and A15-Nb_3_X (X = Al, Ge, Si, Sn) phases and the tetragonal Nb_3_Si [1,3,21]. The Laves phase can be stable in Cr rich alloys and is considered to improve oxidation resistance. The A15 Nb_3_X phase(s) also can be stable in the microstructure depending on concentration(s) of element(s) X and can form during oxidation. The tetragonal Nb_3_Si can be stable or transform to the low temperature αNb_5_Si_3_ via the eutectoid reaction Nb_3_Si → Nb + αNb_5_Si_3_ [2,4,5,23,24,25,26,27,28,29,30].

The Nb_ss_ is the key phase for meeting the fracture toughness property goal but has a negative effect on creep and oxidation when present at a high volume fraction. The toughness of the Nb-0.8Si and (Nb,Ti,Cr,Hf,Si,Ge) solid solutions was reported as 17.7 MPa(m)^1/2^ and ≥28.7 MPa(m)^1/2^ respectively [31,32], more than five and nine times the toughness of unalloyed Nb_5_Si_3_, which is about 3 MPa(m)^1/2^ [21]. The Nb_5_Si_3_ is the key phase for meeting the creep goal but high volume fractions of the silicide decrease the toughness of the in situ composites. The creep exponent of Nb (≈6) [21] is six times that of Nb_5_Si_3_ (≈1) [33]. The low fracture toughness of tetragonal Nb_5_Si_3_ is similar to that of Mo_5_Si_3_ (2–2.5 MPa(m)^1/2^, [34]) and Ti_5_Si_3_ (2.1 MPa(m)^1/2^ [35] and 2.6 MPa(m)^1/2^ [36]). Alloying improved the toughness of Nb_5_Si_3_, which was reported to be 7 MPa(m)^1/2^ and 13 MPa(m)^1/2^ respectively for the (Nb,Ti,Hf,Cr,Fe)_5_(Si,Ge,Al,Sn)_3_ and (Nb,Ti,Hf,Cr)_5_(Si,Ge)_3_ silicides [32]. The compressive fracture strength of Nb_5_Si_3_ was reported to be 670 MPa at 1773 K [31]. The compressive creep rate of arc melted αNb_5_Si_3_ at 1473 K and 69 MPa was 2.23 × 10^−9^ s^−1^ [33] compared with 4 × 10^−8^ s^−1^ of arc melted tetragonal D8_m_ (tI32, W_5_Si_3_-type) Mo_5_Si_3_ [37] and 2 × 10^−5^ s^−1^ of hexagonal D8_8_ (hP16, Mn_5_Si_3_-type) Ti_5_Si_3_ [38].

Table 1 gives available data for the CTE anisotropy of binary (unalloyed) and ternary 5-3 silicides. Data for creep and toughness of binary 5-3 silicides was given above. Developmental Nb-silicide based alloys can have as many as twelve alloying additions, some of which substitute Nb and others Si in Nb_5_Si_3_. For example, refractory metals provide solid solution strengthening to the Nb_ss_ and improve its high temperature strength and simple metal and metalloid element additions improve oxidation. The following composition (at.%) [40.7Nb-12.8Ti-4.7Mo-1.3W-1.5Hf-2.7Cr]-(20.8Si-5.9Ge-4.6Al-5Sn) is an example of a real tetragonal Nb_5_Si_3_ silicide in a developmental Nb-silicide based alloy, where in parentheses are the elements that substitute Si and in square brackets the elements that substitute Nb. There are four sub-lattices in αNb_5_Si_3_ (tI32 (D8_l_), Cr_5_B_3_-type) and it is not known which lattice positions are occupied by the different elements. 

Data about the alloying and properties of Nb_5_Si_3_ is missing in the literature, yet it is crucial for the design of new Nb-silicide based alloys. The motivation for the research presented in this paper was to study the alloying behaviour and properties of Nb_5_Si_3_. The alloying and properties of C14-NbCr_2_ and A15-Nb_3_X phases that are stable in Nb-silicide based alloys will be the subject of another paper.

Recently, it was shown that the alloying of the Nb_ss_ in Nb-silicide based alloys depends on composition weighted differences in electronegativity (Δχ) and an average valence electron concentration (VEC) [22]. Phase stability can be considered in terms of *e*/*a* (an averaged valence of alloying elements in an alloy) and VEC (number of valence electrons per atom filled into the valence band). The former is the parameter in the Hume-Rothery rules and the latter is key to determining the Fermi level in the valence band. The choice between *e*/*a* and VEC depends on the stability mechanism involved [39]. According to Mizutani et al. [39,40], the *e*/*a* is difficult to use as a universal parameter in alloy design because its value cannot be uniquely assigned to a transition metal as it depends on the surrounding environment (the alloying elements in synergy). Instead, VEC is a more important parameter in transition metal alloys. 

In this work, the silicide parameters VEC and Δχ were used to study the alloying and properties of tetragonal Nb_5_Si_3_. One objective was to find out if there are relationships between solvent and solute additions and between the latter and the silicide parameters VEC and Δχ. Another objective was to find out whether changes in properties of tetragonal Nb_5_Si_3_ are related to changes of the silicide parameters VEC and Δχ. 

The structure of the paper is as follows. The effects of alloying on the solubility range of X in (Nb,TM)_5_X_3_ where X = Al + B + Ge + Si + Sn and TM is a transition and/or refractory metal are discussed first, followed by relationships between solutes and their concentrations in Nb_5_Si_3_ and the silicide parameters VEC and Δχ and how alloying influences the hardness of tetragonal Nb_5_Si_3_. The latter is discussed further with the help of the silicide parameter VEC using silicides alloyed with Ge as an example. Finally, the alloying and creep of Nb_5_Si_3_ is discussed with the help of Δχ versus VEC maps. 

## 2. Methodology, Results and Discussion

Available experimental data for tetragonal Nb_5_Si_3_ silicides in developmental Nb-silicide based alloys was used to seek out relationships between the silicide parameters Δχ and VEC, the hardness of tetragonal Nb_5_Si_3_ and changes of the creep of Nb_5_Si_3_ with alloying. For these tasks, it is necessary to know the actual compositions of the Nb_5_Si_3_ silicides in studied alloys [4,5,6,30,41,42,43,44,45,46,47,48] in order to calculate the silicide parameters VEC and Δχ. All the tetragonal Nb_5_Si_3_ silicides studied in this paper were in developmental Nb-silicide based alloys that had been prepared using the same method of arc melting with non-consumable tungsten electrode in an inert atmosphere with water cooled copper crucibles. The phases (Nb_ss_, Nb_5_Si_3_ and others, see introduction) in the cast and heat treated microstructures were identified using XRD (Hiltonbrooks Ltd., Crewe, UK) and JCPDS data (International centre for diffraction data) and quantitative microanalysis [4,5,6,30,41,42,43,44,45,46,47,48]. For the latter, electron probe micro-analysis (EPMA) was used in a JEOL 8600 EPMA (JEOL Ltd., Tokyo, Japan) equipped with energy-dispersive and wavelength-dispersive spectrometers. Standards of high purity elements of Nb, Si and other alloying additions (Al, B, Cr, Ge, Hf, Mo, Si, Sn, Ta, Ti), which had been polished to a finish of 1μm, were used. The operational software was the Oxford Link INCA software (Oxford Instruments plc, Abingdon, UK) that includes the XPP corrections method (matrix correction algorithm to convert k-ratios to element concentrations), which is based on the Rhi-Rho-Z approach. At least 10 analyses for each phase or area of the ingot were performed. The hardness of Nb_5_Si_3_ in the alloys was measured using a Mitutoyo micro-hardness testing machine (Mitutoyo America, Aurora, IL, USA). The load used was 0.1 kg and was applied for 20 s. At least 10 measurements were taken for each phase. The hardness measurements were taken from silicides in bulk microstructures free of contamination by interstitials and with similar grain sizes. The data for the compressive creep of Nb_5_Si_3_ was from the references [33,49], where the experimental details for the creep experiments were given. No new experimental data were created during the course of this study.

The parameter VEC was calculated using [VEC]_intermetallic_ = ∑*_i_*^n^*C_i_*(*VEC*)*_i_*, where *C_i_* and (*VEC*)*_i_* respectively are the concentration (at.%) and VEC of element *i* in the silicide. For the Nb_5_Si_3_ silicide [Δχ]_silicide_ = ∑_i_^m^*c_i_*(χ*_<_*_Nb*>i*_) − ∑_i_^z^ *< κ_i_*(*χ_<_*_Si*>i*_), where *c_i_* and *χ_<_*_Nb*>i*_ respectively are the concentration (at.%) and Pauling electronegativity of Nb and element *i* substituting Nb in the silicide and *κ_i_* and *χ_<Si>i_* respectively are the concentration (at.%) and Pauling electronegativity of Si and element *i* substituting Si in the silicide. Data for electronegativity and VEC was from the same sources as in [22].

The unalloyed (binary) tetragonal αNb_5_Si_3_ and the B containing tetragonal Nb_5_Si_3_ are also known as the T1 and T2 silicides respectively and both have the Cr_5_B_3_ as their prototype. In Nb-silicide based alloys, the Nb in Nb_5_Si_3_ can be substituted by other transition and/or refractory metals, e.g., Cr, Hf, Mo, Ta, Ti, W and the Si by other simple metals and metalloids, e.g., Al, B, Ge and Sn [4,5,6,30,41,42,43,44,45,46,47,48]. The solubilities of most of these elements depend on other alloying additions, in particular Ti. An objective of this work was to find out if solvent and solute concentrations in the Nb_5_Si_3_ are related and whether the concentrations of solute additions in the silicide depend on the parameters VEC and Δχ. The alloying of Nb_5_Si_3_ can stabilise the high temperature tetragonal βNb_5_Si_3_ and/or the low temperature tetragonal αNb_5_Si_3_ and/or the hexagonal γNb_5_Si_3_ in the microstructure of Nb-silicide based alloys and can change the mechanical properties and oxidation of these silicides. Another objective of this work was to find out how properties of tetragonal Nb_5_Si_3_ are associated with changes of the parameters VEC and Δχ.

In Ti containing Nb-silicide based alloys, Ti rich Nb_5_Si_3_ can form in the cast microstructure owing to the partitioning behaviour of Ti [4,22]. The Ti rich Nb_5_Si_3_ tends to persist in the microstructure after heat treatment. In the SEM and EPMA the Ti rich Nb_5_Si_3_ is recognised by its different contrast in the microstructure under back scatter electron imaging conditions [4].

First it will be shown that the available microanalysis data can be used to find out how the solubilities of elements that substitute Nb and Si in Nb_5_Si_3_ are related. The actual chemical composition data for Nb_5_Si_3_ in Nb-silicide based alloys [4,5,23,24,25,26,27,28,29,30,41,42,43,44,45,46,47,48] showed that the solubilities of the elements that substitute Nb in tetragonal Nb_5_Si_3_ are Cr ≤ 2.9 at.%, Hf ≤ 10.6 at.%, Mo ≤ 1.9 at.%, Ta ≤ 6.3 at.%, Ti ≤ 32.8 at.% and W ≤ 1.9 at.% and that the solubility range of Ti in Nb_5_Si_3_ depends on the concentration of Ti in the alloy. For KZ5 type alloys [4], i.e., for alloys where other transition metals and simple metals and metalloid elements are added to the nominal composition Nb-24Ti-18Si-5Al-5Cr (at.%), which is the composition of the alloy KZ5, the Ti solubility range is 17.1 < Ti < 24.2 at.% and 22.2 < Ti < 32.8 at.% for normal and Ti rich Nb_5_Si_3_ respectively.

The actual chemical composition data for Nb_5_Si_3_ in Nb-silicide based alloys [4,5,23,24,25,26,27,28,29,30,41,42,43,44,45,46,47,48] showed that the solubilities of the elements that substitute Si in Nb_5_Si_3_ in Nb-silicide based alloys are Al ≤ 5.2 at.%, B ≤ 10.4 at.%, Ge ≤ 8.4 at.% and Sn ≤ 3.8 at.% with 33.6 < X < 41.6 at.% (X = Al + B + Ge + Si + Sn), compared with 37.5 < Si < 40.5 at.% for unalloyed (binary) Nb_5_Si_3_ [2]. The value of X does not differ significantly between the normal silicide and Ti rich Nb_5_Si_3_ and is 33.9 < X < 40.9 at.% and 33.6 < X < 41.6 at.% respectively. However, the range of X values would suggest that the solubility range of tetragonal Nb_5_Si_3_ opens up, or closes down and/or shifts upon alloying, primarily towards the Nb side. These changes are accompanied by changes in the values of the parameters VEC and Δχ.

The values of the parameters VEC and Δχ of the Nb_5_Si_3_ silicide that were calculated as described above are in the ranges 4.11 < VEC < 4.45 and 0.103 < Δχ < 0.415 respectively. The silicide parameter VEC falls outside the range of VEC values of the Nb_ss_ [22]. The range of the values of the silicide parameter Δχ is wider that those of the Nb_ss_ [22] and there is a gap in silicide Δχ values in the range 0.13 < Δχ < 0.15, which falls within the 0.13 < Δχ < 0.18 gap for the Δχ of the Nb_ss_ [22]. However, in the case of the tetragonal Nb_5_Si_3_ silicide, the aforementioned gap is observed only for B containing Nb_5_Si_3_ (i.e., for alloyed T2).

Table 2 shows the solubility range of Si for binary Nb_5_Si_3_ [2] and the solubility range of X in (Nb,TM)_5_X_3_ silicides in KZ5 type alloys, where X = Al + B + Ge + Si + Sn and transition and refractory metals are represented by TM. Data for chemical compositions of alloys in Table 2 can be found in the references [4,6,41,50,51]. For each alloy in Table 2, the corresponding values of the parameters VEC and Δχ of Nb_5_Si_3_ were calculated as described above and are given for the cast and heat treated conditions. In the binary (unalloyed) Nb_5_Si_3,_ the Si concentration varies from 37.5 at.%, for which Δχ = 0.288 and VEC = 4.63, to 40.5 at.% (Δχ = 0.183, VEC = 4.6). In the alloy KZ5 [4] the (Nb,Ti,Cr)_5_(Si,Al)_3_ has Al + Si in the range 35.3 < X < 36.4 at.% (X = Al + Si) and the values of the parameters change from Δχ = 0.363, VEC = 4.41 (cast) to Δχ = 0.322 and VEC = 4.42 (heat treated). With the addition of 6Ta in the alloy KZ6 [6], the (Nb,Ti,Cr,Ta)_5_(Si,Al)_3_ has Al + Si in the range 36.7 < X < 38.7 at.% and the values of the parameters change from Δχ = 0.309, VEC = 4.38 (cast) to Δχ = 0.236 and VEC = 4.41 (heat treated). With the addition of 5B the (Nb,Ti,Cr)_5_(Si,Al,B)_3_ has X = Al + B + Si in the range 36.7 < X < 39 at.% and the values of the parameters change from Δχ = 0.303, VEC = 4.27 (cast) to Δχ = 0.224 and VEC = 4.31 (heat treated).

The data in Table 2 shows that individually the transition metals Hf, Mo and Ta and the elements B, Ge and Sn (when added to the alloy KZ5) shift the solubility range X of the (Nb,TM)_5_X_3_ silicide towards Nb, with Hf and Ge having the strongest effect. Boron in synergy with Hf or Mo or Sn opens up the solubility range beyond 40.5 at.%. It should be noted that for each alloy a shift towards higher X concentrations is accompanied by a decrease in the value of Δχ. However, the change of the parameter VEC (meaning increase or decrease) depends on the alloying addition(s), for example when 5 at.% Hf is added to the alloy KZ5 the parameter VEC decreases but when 6 at.% Ta is added the parameter VEC increases.

**Figure 1:**
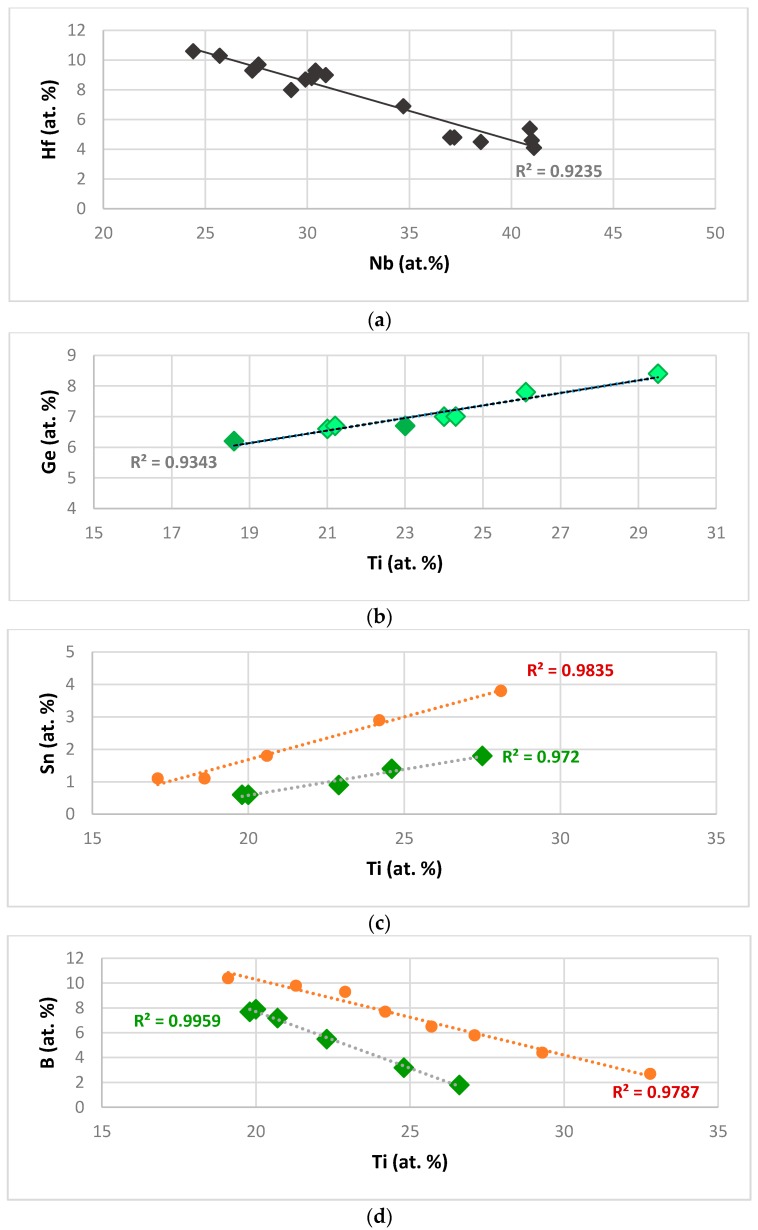
Relationships between alloying elements in Nb_5_Si_3_. (**a**) Shows the relationship between Hf (at.%) and Nb (at.%) in Nb_5_Si_3_. (**b**–**d**) show the relationships respectively between Ge, Sn and B (at.%) and Ti (at.%) in Nb_5_Si_3_. In (**b**) the dark green diamonds are for alloys with Ge and Sn. In (**c**) and (**d**) the green diamonds are for alloys with B and with no Mo, respectively.

The availability of data about the actual chemical composition of alloyed Nb_5_Si_3_ makes it possible to study the alloying of tetragonal Nb_5_Si_3_. The Si concentration in the silicide decreases with increasing Ti concentration in the silicide and the Cr and Al concentrations increase with increasing Ti concentration. Figure 1a shows that the Hf concentration in Nb_5_Si_3_ decreases linearly with increasing Nb concentration in the silicide. The linear fit of the data is better in the Hf versus Nb plot (R^2^ = 0.9235) compared with the Ti versus Nb plot (not shown, R^2^ = 0.8095) that indicates that the Hf concentration in the Nb_5_Si_3_ increases with its Ti concentration. This would suggest that the Hf concentration in the Nb_5_Si_3_ depends on both Nb and Ti in the Nb_5_Si_3_. Figure 1b shows that the concentration of Ge in Nb_5_Si_3_ increases with that of Ti. Note that in Figure 1b the data for Ge and Sn containing Nb_5_Si_3_ (darker diamonds) falls on the same line as that for Nb_5_Si_3_ in Ge containing alloys with no B and Sn. The dependence of the concentration of Sn and B on that of Ti in Nb_5_Si_3_ is shown in Figure 1c,d respectively. The different sets of data in each part are not for normal Nb_5_Si_3_ and Ti rich Nb_5_Si_3_ but for different solutes in the silicide as indicated in the figure caption. The Figure 1c,d would suggest that the solubilities of B and Sn in Nb_5_Si_3_ depend strongly on the other elements that are present in the alloy. In all parts of Figure 1 the linear fit of data, shown by the R^2^ values, is very good.

**Figure 2:**
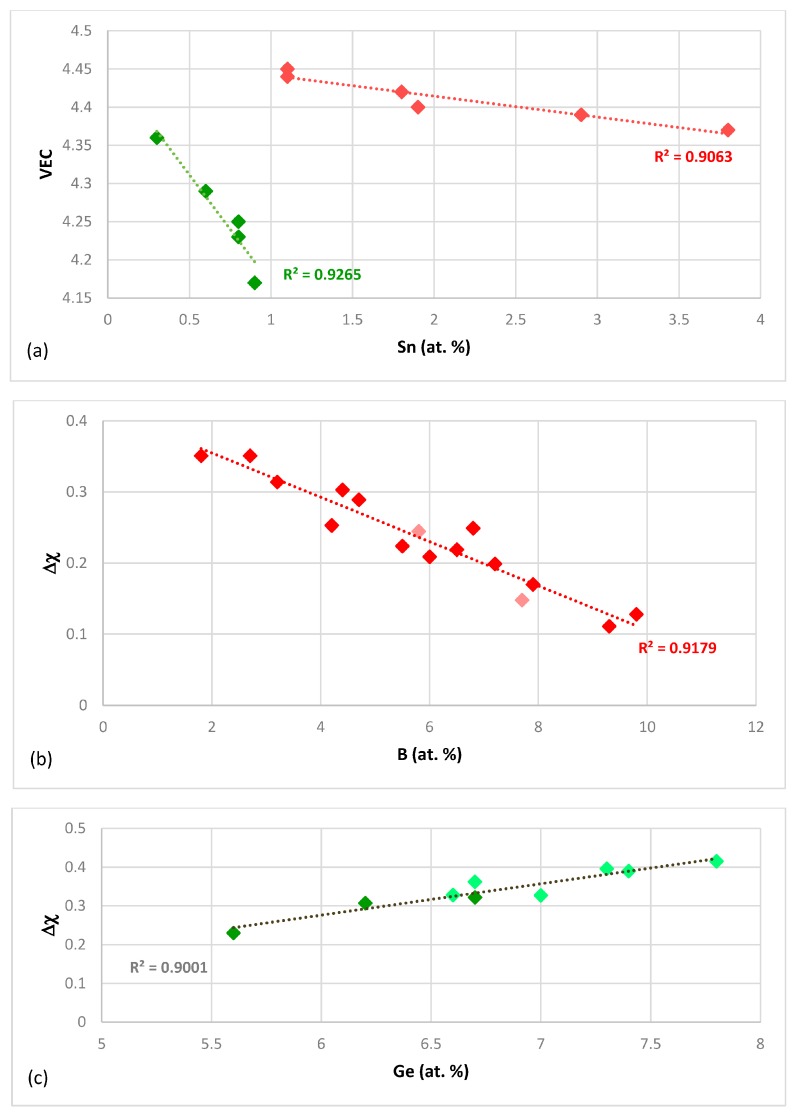
Relationships between the silicide parameters VEC and Δχ and solute additions that substitute Si in Nb_5_Si_3_. (**a**) Shows the relationship between VEC and Sn (at.%) in Nb_5_Si_3_. The red diamonds are for alloys that contain Ge and the green diamonds for alloys that contain B—(**b**,**c**) show the relationships between Δχ and B (at.%) (**b**) or Ge (at.%) (**c**) in Nb_5_Si_3_. In (**b**) the light red diamonds are for alloys with B and Sn and in (**c**) the dark green diamonds are for alloys with Ge and Sn.

What can be learned about the alloying of Nb_5_Si_3_ from the silicide parameters VEC and Δχ? The silicide parameter VEC can separate the alloying behaviour of Hf in the normal and Ti rich Nb_5_Si_3_. The value of the silicide parameter VEC decreases with increasing Hf concentration in Nb_5_Si_3_ but there is no strong relationship (the R^2^ value is low). However, the silicide parameter VEC can better describe the alloying behaviour of Sn in Nb_5_Si_3_ (Figure 2a), which is shown to depend strongly on the elements that are present in the alloy (as was the case in Figure 1c), with B having a strong effect on the change of VEC with Sn concentration in the silicide, compared with that of Ge. 

The silicide parameter Δχ also can separate the alloying behaviour of Hf in the normal Nb_5_Si_3_ and Ti rich Nb_5_Si_3_ but the data falls in two distinct parts with no strong relationship (the R^2^ value is low). When the data for Sn is considered, the silicide parameter Δχ can separate the data into two groups for B and Sn and Ge and Sn containing alloys but there is no good linear fit of the data compared with the silicide parameter VEC (Figure 2a). The silicide parameter Δχ also can describe the alloying behaviour of B in Nb_5_Si_3_ (Figure 2b) well but cannot separate the effect of transition metal addition in the alloy, which was demonstrated in Figure 1d. The parameter Δχ decreases with increasing B concentration in Nb_5_Si_3_ (Figure 2b). The alloying of Nb_5_Si_3_ with Ge also can be described well by Δχ, which increases with Ge concentration in the alloy (Figure 2c). Note that the trends in Figure 2b,c regarding the changes of the silicide parameter Δχ with B and Ge concentration in Nb_5_Si_3_ are the same with the trends in the change of the concentrations of these elements with Ti in Nb_5_Si_3_, shown in Figure 1c,d respectively. This is not the case for the silicide parameter VEC and the concentration of Sn in Nb_5_Si_3_ (Figure 1c and Figure 2a).

**Figure 3:**
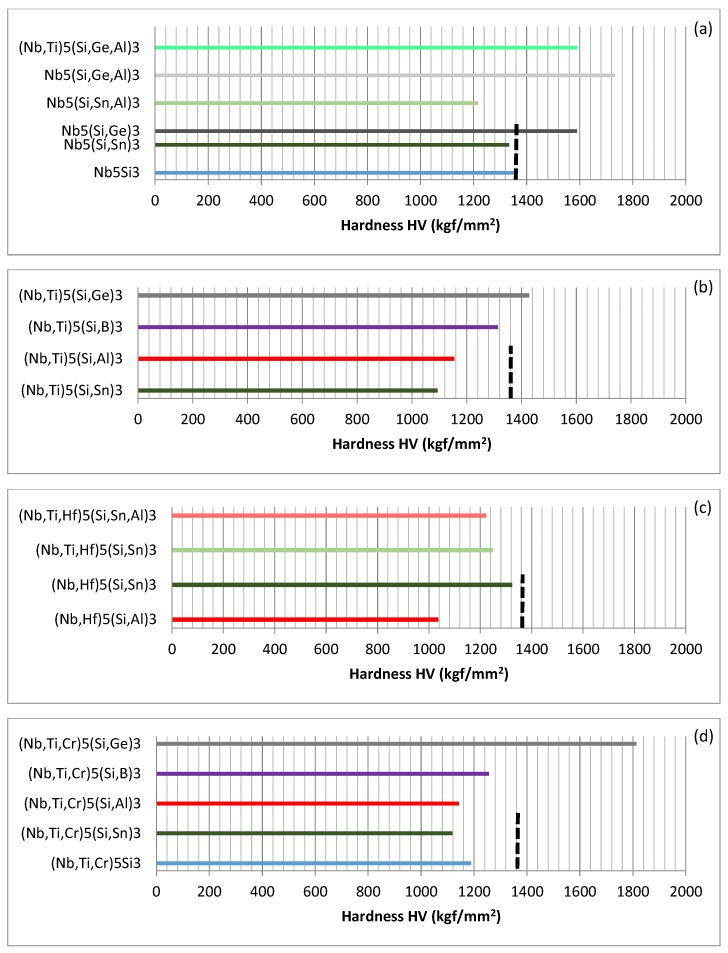
Bar charts showing the average Vickers hardness (HV) of tetragonal Nb_5_Si_3_. The vertical dashed lines indicate the hardness of binary (unalloyed) Nb_5_Si_3_. In the parts (**a**), (**b**), (**c**) and (**d**) the data is from [44,45,50], [29,46], [45,51], [46,47], respectively.

Links between alloying and properties will now be considered. The effects of alloying on the hardness of tetragonal Nb_5_Si_3_ are shown in Figure 3, which shows the data for the average Vickers hardness (HV) of tetragonal Nb_5_Si_3_ silicide, where Nb and Si are substituted by different elements. The data in Figure 3 shows that Ge increases significantly the hardness of Nb_5_Si_3_ compared with Sn, which hardly changes the hardness (see Figure 3a). The effect of Al on the hardness of Nb_5_Si_3_ depends on the other element that substitute Si in the silicide. Aluminium has a strong negative and positive effect when it is in synergy with Sn or Ge respectively (see Figure 3a). Comparison of the data for Nb_5_(Si,Ge,Al)_3_ with that for (Nb,Ti)_5_(Si,Ge,Al)_3_ in Figure 3a suggests that the substitution of Nb by Ti decreases the hardness of the 5-3 silicide. This cannot be confirmed for the ternary silicide, because, to the author’s knowledge, there is no experimental data available for the hardness of (Nb,Ti)_5_Si_3_. 

The effect of alloying with Ti on the Young’s modulus is shown in Table 3, where data for unalloyed αNb_5_Si_3_, βNb_5_Si_3_ and alloyed α(Nb,Ti)_5_Si_3_ and β(Nb,Ti)_5_Si_3_ with 12.5 at.% Ti is given together with the Young’s moduli of other TM tetragonal 5-3 silicides. In [19,42] it was shown that (i) the βNb_5_Si_3_ has lower Young’s modulus E, shear modulus G and G/B ratio (B is the bulk modulus) compared with the αNb_5_Si_3_ and; (ii) that substitution of Nb by Ti increases and decreases the E, G and G/B respectively for the αNb_5_Si_3_ and βNb_5_Si_3_. 

The effect of Ti on the hardness of (Nb,Ti)_5_Si_3_ can be deduced using data for E, G, the G/B ratio and Poisson’s ratio ν for unalloyed and Ti alloyed tetragonal Nb_5_Si_3_ silicides from [19,42] (see Table 4). The calculations showed that the hardness of βNb_5_Si_3_ is lower than that of αNb_5_Si_3_ and alloying with Ti respectively decreases and increases the hardness of these silicides. The hardness of βNb_5_Si_3_ (HV = 1286) that was calculated using data for the calculated G/B ratio is closer to the experimental value for unalloyed Nb_5_Si_3_ (HV = 1360). Table 4 also gives data for the calculated hardness of the unalloyed hexagonal Ti_5_Si_3_, which is higher than the measured hardness of unalloyed Ti_5_Si_3_ (1154 ± 55 HV [56]). The calculations indicate that alloying Nb_5_Si_3_ with Ti decreases the hardness of β(Nb,Ti)_5_Si_3_ only.

The hardness values of Nb_5_Si_3_ where Nb is substituted by Ti only and Si by Al or B or Ge or Sn are compared in Figure 3b. The data provides further support that Ti has a negative effect on the hardness and also shows that the synergy of Si, Sn and Ti has the strongest negative effect while that of Ge, Si and Ti has the weakest negative effect, compared with the data in Figure 3a, with the hardness gradually increasing as the Si is substituted by Sn, Al, B and Ge. The hardness of (Nb,Ti)_5_(Si,Ge)_3_ is slightly higher than that of the binary (unalloyed) Nb_5_Si_3_.

When Hf substitutes Nb and Al or Sn substitutes Si, the synergy of Al and Hf and Hf and Sn respectively has a stronger negative and positive effect on the hardness compared with that of Al and Ti and Sn and Ti (Figure 3b,c). When both Ti and Hf substitute Nb and Sn substitutes Si the hardness decreases slightly, compared with (Nb,Hf)_5_(Si,Sn)_3_ and there is a further small decrease in hardness when both Al and Sn substitute Si (Figure 3c). Notice that all the 5-3 silicides in Figure 3c have lower hardness than that of the binary (unalloyed) Nb_5_Si_3_.

The effect of the substitution of Nb by Cr and Ti on the hardness of Nb_5_Si_3_ is shown in Figure 3d. When only Nb is substituted in Nb_5_Si_3_ the hardness decreased (compared with the unalloyed Nb_5_Si_3_) and there is further decrease when Si is substituted by Al and Sn and the effect of Al or Sn is essentially the same (compared with (Nb,Ti,Cr)_5_Si_3_). The hardness increases as Si is substituted by Al and B and increases significantly when Si is substituted by Ge (compared with (Nb,Ti,Cr)_5_(Si,Sn)_3_). In Figure 3d, only the (Nb,Ti,Cr)_5_(Si,Ge)_3_ has hardness higher than that of the unalloyed Nb_5_Si_3_ and its hardness is the highest of all the 5-3 silicides shown in Figure 3.

**Figure 4:**
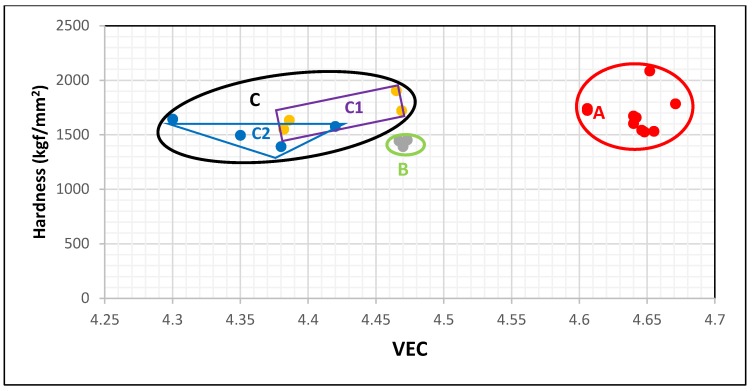
Vickers hardness of tetragonal Nb_5_Si_3_ versus silicide parameter VEC in alloyed Nb_5_Si_3_ with Ge but with no B, Sn, Mo, Ta or W. The data is represented by filled circles. The data shown in red colour is for Nb_5_(Si,Ge)_3_, (Nb,Cr)_5_(Si,Ge)_3_, Nb_5_(Si,Ge,Al)_3_, the data shown in green colour is for (Nb,Ti)_5_(Si,Ge)_3_, the data shown in purple colour is for Nb_5_Si_3_ alloyed with Ge and with Ti + Cr (i.e., (Nb,Ti,Cr)_5_(Si,Ge)_3_) or with Ti + Al and the data shown in blue colour is for Nb_5_Si_3_ alloyed with Ge and with Ti + Al + Cr or with Ti + Hf + Al + Cr. For areas A, B, C, the rectangle C1 and the triangle C2 see text.

In this paper, the data for Nb_5_Si_3_ alloyed with Ge was chosen in order to demonstrate how alloying changes the hardness of Nb_5_Si_3_ and how the change of hardness can be understood using the silicide parameter VEC. The data in Figure 4 falls in three groups represented by the red, green and black areas that are labelled A, B and C. The data for the silicides Nb_5_(SiGe)_3_, (Nb,Cr)_5_(Si,Ge)_3_ and Nb_5_(Si,Ge,Al)_3_ is in area A. When Nb is substituted only by Ti and Si only by Ge the data shifts towards lower VEC and hardness values to area B, which contains the data for (Nb,Ti)_5_(Si,Ge)_3_. The individual addition of Al or Cr to the silicide shifts the data upwards (higher hardness but lower VEC) to the rectangular C1 in area C (black ellipse), which has the data for (Nb,Ti,Cr)_5_(Si,Ge)_3_ and (Nb,Ti)_5_(Si,Ge,Al)_3_. The substitution of Si by Al shifts VEC and hardness to lower values compared with Cr. The simultaneous presence of Al and Cr in the silicide shifts the data towards lower VEC to the triangle C2 in area C. Thus, the map of silicide hardness versus silicide parameter VEC clearly differentiates the role played by Ti in the hardness of Nb_5_Si_3_ alloyed with Ge and with no B, Sn, Mo, Ta, or W additions. When no Ti is present in the silicide the hardness exceeds 1500 HV and the silicide parameter VEC is higher than 4.6. The addition of Ti causes VEC to decrease to values below 4.48 and this is accompanied by a shift of the hardness to lower values. There is a gap in VEC values between 4.6 and 4.48 for Nb_5_Si_3_ alloyed with Ge. 

**Figure 5:**
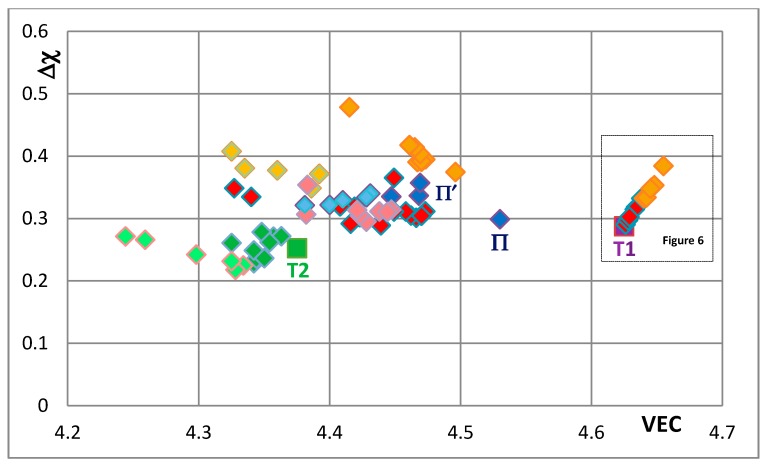
Δχ versus VEC map of all studied Nb_5_Si_3_ that shows the effect of the substitution of Nb by Ti and Cr and Si by Al, B, Sn or Ge on the position of the silicide in the map. The unalloyed Nb_5_Si_3_ (T1) and the Nb_5_(Si,B)_3_ (T2) silicides are shown respectively by the purple and green squares. The symbol Π is for the (Nb,Ti)_5_Si_3_. Silicides where Si is substituted by B, Ge and Sn are shown by dark green, dark gold and dark red diamonds, respectively. Silicides where Si is substituted by Al and B or Ge or Sn are shown by lighter green, lighter gold and lighter red diamonds, respectively. Silicides that do not contain B or Ge or Sn are shown by dark blue diamonds. Silicides where Si is substituted by Al but not B, Ge or Sn are shown by lighter blue diamonds. For inset see Figure 6. For the data represented by Π and Π’ see text.

**Figure 6:**
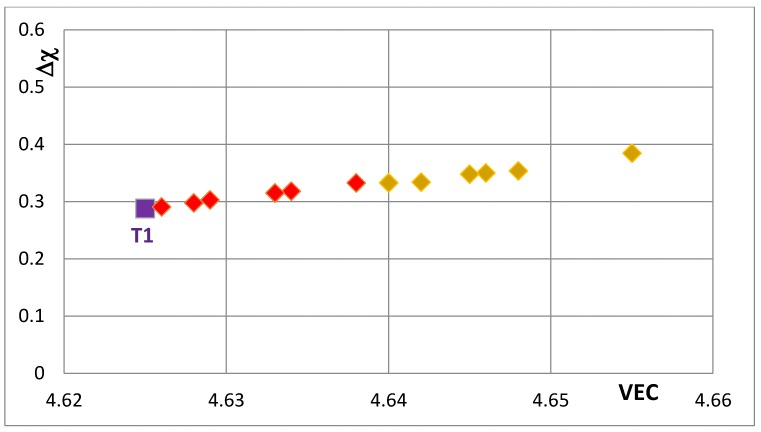
Δχ versus VEC map of Nb_5_Si_3_ that shows the effect of the substitution of Si by Sn or Ge on the position of the silicide in the map. The Nb_5_Si_3_ (T1), Nb_5_(Si,Sn)_3_ and Nb_5_(Si,Ge)_3_ are shown respectively by purple square and by red and gold diamonds.

**Figure 7:**
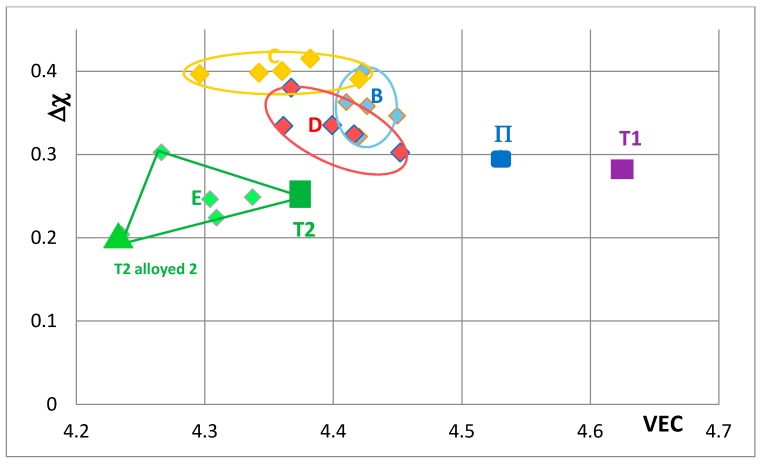
Δχ versus VEC map of unalloyed and alloyed Nb_5_Si_3_, where T1 = Nb_5_Si_3_, Π = (Nb,Ti)_5_Si_3_, T2 = Nb_5_(Si,B)_3_, T2 alloyed2 = (Nb,Ti,Cr,Hf)_5_(Si,Al,B)_3_, area B = (Nb,Ti,Cr)_5_(Si,Al)_3_, area C = (Nb,Ti,Cr)_5_(Si,Al,Ge)_3_, area D = (Nb,Ti,Cr)_5_(Si,Al,Sn)_3_ and area E = (Nb,Ti,Cr)_5_(Si,Al,B)_3_. The data is from KZ5 type alloys with nominal compositions Nb-24Ti-18Si-5Al-5Cr + 5X, where X = B, Ge, Sn. Average positions in areas B to E are indicated by data point closest to letter (see text). For the compositions of Π, T2 and T2 alloyed2 see text.

**Figure 8:**
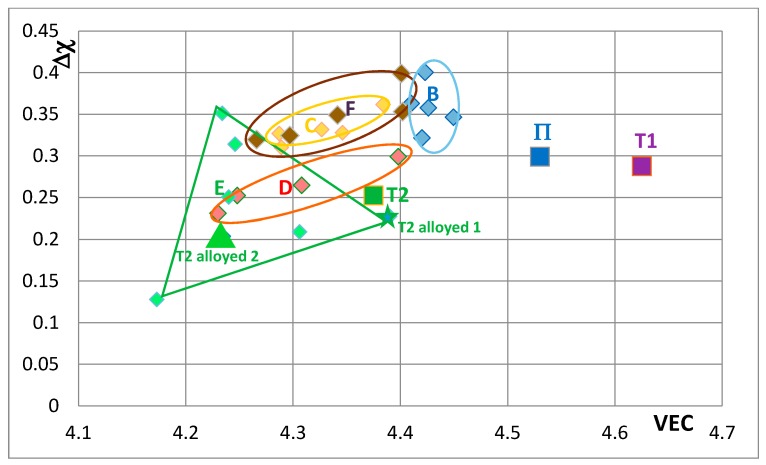
Δχ versus VEC map of unalloyed and alloyed Nb_5_Si_3_ that shows the effect of the substitution of Nb by Hf (and Ti and Cr), where T1 = Nb_5_Si_3_, Π = (Nb,Ti)_5_Si_3_, T2 = Nb_5_(Si,B)_3_, area B = (Nb,Ti,Cr)_5_(Si,Al)_3_, area C = (Nb,Ti,Cr,Hf)_5_(Si,Al,Ge)_3_, area D = (Nb,Ti,Cr,Hf)_5_(Si,Al,Sn)_3_, area E = (Nb,Ti,Cr,Hf)_5_(Si,Al,B)_3_ and area F = (Nb,Ti,Cr,Hf)_5_(Si,Al)_3_. The data is from KZ5 type alloys with nominal compositions Nb-24Ti-18Si-5Al-5Cr + 5Hf + 5X, where X = B, Ge, Sn. Average positions in areas B to F are indicated by data point closest to letter (see text). For compositions of Π, T2 and T2 alloyed1 andT2 alloyed2 see text.

**Figure 9:**
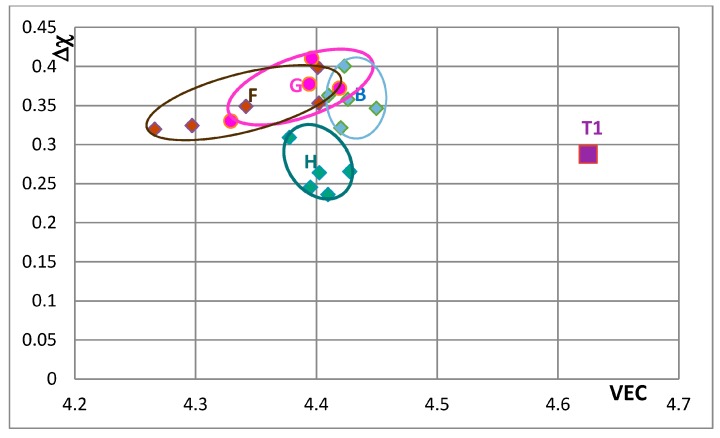
Δχ versus VEC map of unalloyed and alloyed Nb_5_Si_3_ that shows the effect of the substitution of Nb by a transition metal or refractory metal addition, where T1 = Nb_5_Si_3_, area B = (Nb,Ti,Cr)_5_(Si,Al)_3_, area F = (Nb,Ti,Cr,Hf)_5_(Si,Al)_3_, area G = (Nb,Ti,Cr,Mo)_5_(Si,Al)_3_ and area H = (Nb,Ti,Cr,Ta)_5_(Si,Al)_3_. The data is from KZ5 type alloys with nominal compositions Nb-24Ti-18Si-5Al-5Cr + 5X, where X = Hf, Mo, Ta. Average positions in areas B and F to H are indicated by data point closest to letter (see text).

**Figure 10:**
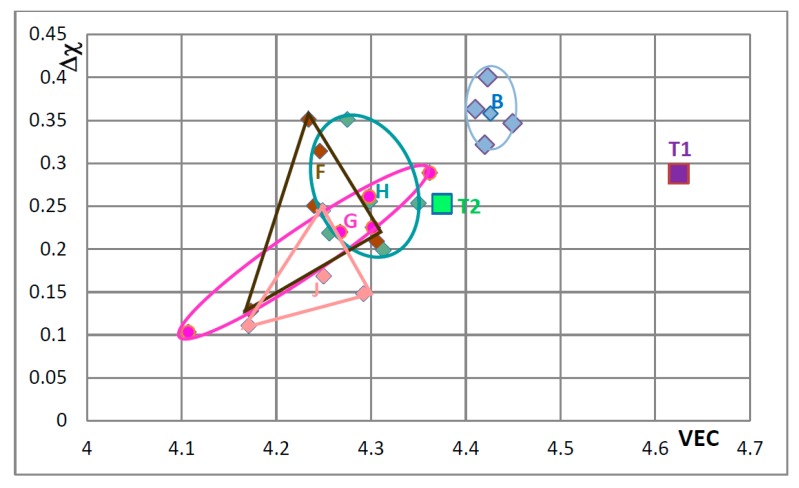
Δχ versus VEC map of unalloyed and alloyed Nb_5_Si_3_ that shows the effect of the substitution of Nb by a transition metal or refractory metal addition, where T1 = Nb_5_Si_3_, T2 = Nb_5_(Si,B)_3_, area B = (Nb,Ti,Cr)_5_(Si,Al)_3_, area F = (Nb,Ti,Cr,Hf)_5_(Si,Al,B)_3_, area G = (Nb,Ti,Cr,Mo)_5_(Si,Al,B)_3_, area H = (Nb,Ti,Cr,Ta)_5_(Si,Al,B)_3_ and area J = (Nb,Ti,Cr)_5_(Si,Al,B,Sn)_3_. The data is from KZ5 type alloys with nominal compositions Nb-24Ti-18Si-5Al-5Cr + 5X, where X = Hf, Mo, Ta, Sn. Average positions in areas B and F to J are indicated by data point closest to letter (see text).

**Figure 11:**
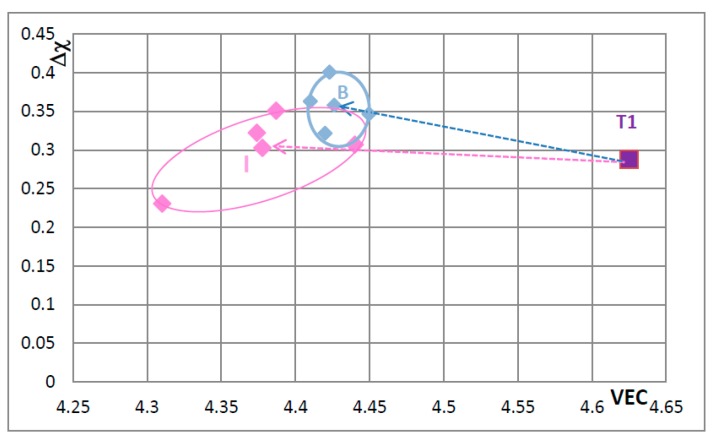
Δχ versus VEC map of unalloyed and alloyed Nb_5_Si_3_ to show the “direction of change” of the position of the silicide in the map with alloying. T1 is Nb_5_Si_3_ and the areas B and I have data for (Nb,Ti,Cr)_5_(Si,Al)_3_ and (Nb,Ti,Cr)_5_(Si,Al,Ge,Sn)_3_, respectively. The data is from KZ5 type alloys with nominal compositions Nb-24Ti-18Si-5Al-5Cr + X, where X = 5Ge + 5Sn. Average positions in areas B and F to H are indicated by data point closest to letter (see text). For arrows see text.

The effects of alloying on properties of Nb_5_Si_3_ also can be demonstrated using maps of the silicide parameters VEC and Δχ and the available data for the creep of unalloyed and alloyed Nb_5_Si_3_. Silicide maps are shown in the Figure 5, Figure 6, Figure 7, Figure 8, Figure 9, Figure 10 and Figure 11. Note that the data in the Figure 7, Figure 8, Figure 9, Figure 10 and Figure 11 is for different alloys. Figure 5 is the map for all the tetragonal Nb_5_Si_3_ silicides in the studied developmental alloys. When Si is substituted by Ge or Sn, the values of the silicide parameters VEC and Δχ increase but the opposite is the case when B substitutes Si (see Figure 5 and Figure 6 and compare the positions of T1 and T2—the composition of the silicide shown by T2 in Figure 5 is 62.5Nb-12.5Si-25B) or Ti substitutes Nb (see Π in Figure 5, which corresponds to the silicide 53Nb-10Ti-37Si [49]) and the concentration of Ti in the silicide is increased (see Π’ in Figure 5 that corresponds to the silicide 46.8Nb-17.4Ti-35.8Si). When both Nb and Si are substituted the values of the silicide parameter VEC decrease further (all data shifts to the left of Π) and Ge and B have the strongest effect on the silicide parameter Δχ with the former increasing and the latter decreasing Δχ (compared with T1) while the effect of Sn depends on alloying additions. When Si is substituted only by Al the silicide parameter VEC decreases further and there is a slight reduction of the value of the silicide parameter Δχ. When Al is substituting Si, the shift towards lower VEC values is increased only for the silicides where Al is simultaneously present with B or Ge but this is not the case when Al and Sn are simultaneously present in the silicide.

Figure 7, Figure 8, Figure 9, Figure 10 and Figure 11 show how the position of Nb_5_Si_3_ changes with alloying additions in the VEC versus Δχ silicide maps. Note differences in the VEC and Δχ axes compared with Figure 5 and Figure 6 and differences in the VEC axes between Figure 7, Figure 8, Figure 9, Figure 10 and Figure 11. The data used in these maps is for normal and Ti rich Nb_5_Si_3_ in cast and heat treated alloys. In Figure 7, Figure 8, Figure 9, Figure 10 and Figure 11 the unalloyed Nb_5_Si_3_ is shown as T1. The alloying additions in Nb_5_Si_3_ are Cr, Hf, Mo, Ta, which substitute Nb and Al, B, Ge and Sn, which substitute Si. Substituting Nb with Ti and Cr and Si with Al shifts the silicide in area B, meaning that the normal Nb_5_Si_3_ and Ti rich Nb_5_Si_3_ in the cast and heat treated alloy “moves” in this area as the concentrations of Al, Cr and Ti in the Nb_5_Si_3_ change. Area B is included in Figure 7, Figure 8, Figure 9, Figure 10 and Figure 11 to show how alloying changes the position of the Nb_5_Si_3_ in the maps. 

Figure 7 shows changes caused by the substitution of Si with B, Ge and Sn. In this figure, the Nb in the silicide is substituted by Ti and Cr. The Nb_5_Si_3_ “shifts” from area B to areas C, D and E in Figure 7, when Ge or Sn or B is present in the alloy. Note that the Nb_5_Si_3_ alloyed with B occupies the distinctly different area E. In Figure 8 the effect of substituting Nb with Hf (and Ti and Cr) and Si with B, Ge and Sn in Nb_5_Si_3_ is shown. The silicide shifts from area B to areas C to F. The substitution of Nb by Cr, Hf and Ti shifts the silicide from area B to area F. When Si in the silicide is substituted by Ge, area C (silicide with Ge and Hf) is entirely within the area F. This however is not the case when Si in the silicide is substituted by Sn in area D, which is for silicides with Hf and Sn. Area D spreads into area E (silicides with B and Hf). Note that with the addition of Hf area E expands towards higher Δχ and lower VEC values. The compositions of the alloyed silicides indicated as T2 alloyed1 and T2 alloyed2 respectively were 38.5Nb-16Ti-6Hf-1Cr-37Si-1Al-0.5B and 41.5Nb-13Ti-3Hf-4Cr-12.5Si-25.5B-0.5Al [49].

In Figure 9 the effect of substituting Nb with other transition metals such as Hf and Mo and Ta and Si only with Al is considered in order to show and compare the effects of Mo and Ta in comparison with the effect of Hf. The Nb_5_Si_3_ alloyed with Ta occupies its own area (H), which is separate from area B. The silicide alloyed with Mo falls almost entirely in area F, which is the same as area F in Figure 8. In Figure 10 the substitutions Si with B and Sn and Nb with Hf or Mo or Ta are considered to show the effect of the simultaneous presence of B with each of the other elements. The addition of B causes a significant change in area F (compare Figure 8, Figure 9 and Figure 10), area H expands (compare Figure 9 and Figure 10) and area G shifts to lower VEC and Δχ values (compare Figure 9 and Figure 10). In Figure 10 the silicides that contain B occupy the area of the map defined by Δχ and VEC with values less than about 0.35 and 4.362 respectively. The higher value of Δχ should also be noted in Figure 8. The simultaneous presence of B and Sn has the strongest effect (compare positions of areas B and J in the map). Figure 11 shows that when Ge and Sn are simultaneously present in the alloy area B shifts to area I, which in the map occupies a position similar to but larger than area D in the Figure 8, that parts of areas B and I overlap and that Δχ is below 0.35.

In Figure 7, Figure 8, Figure 9, Figure 10 and Figure 11 the “average” positions of the Nb_5_Si_3_ in the areas B to I are indicated by the data point that is closest to the letter of the area. One could use an arrow to link the T1 with the average in each area to show “the direction of change” in the map with specific alloying addition(s). To avoid crowding the maps with extra lines, the “direction of change” is demonstrated only in Figure 11, where T1 is connected with the average positions in areas B and I. It is noted that the average Δχ value of the Nb_5_Si_3_ changed very little compared with that of the unalloyed silicide (T1) when Ge and Sn were simultaneously present in the silicide. 

The shift of the position of the Nb_5_Si_3_ in the VEC versus Δχ maps in Figure 5, Figure 6, Figure 7, Figure 8, Figure 9, Figure 10 and Figure 11—when Nb and Si of the silicide were substituted by alloying additions in Nb-silicide based alloys and the change of the composition of the silicide as the alloy microstructure evolved following exposure to high temperature—should be accompanied with changes of the properties of the 5-3 silicide. These changes affect creep and oxidation of the alloys. This will be discussed in a separate paper.

**Figure 12:**
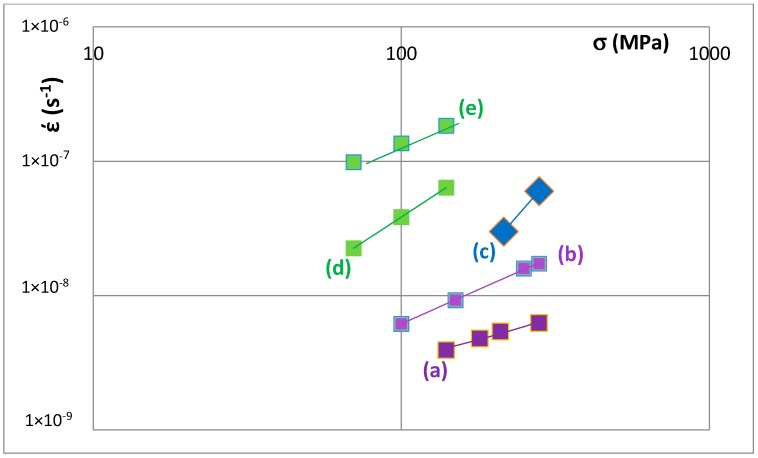
Norton plot for 1473 K to show creep rate $\dot{\varepsilon }$ (s^−1^) versus stress σ (MPa) for tetragonal (**a**) αNb_5_Si_3_ [34]; (**b**) αNb_5_Si_3_ [50] and (**c**–**e**) alloyed Nb_5_Si_3_. (**c**) is for Π = (Nb,Ti)_5_Si_3_ (see text) (**d**) is for T2 alloyed1 = (Nb,Ti,Cr,Hf)_5_(Si,Al,B)_3_ (see text) and (**e**) is for T2 alloyed2 = (Nb,Ti,Cr,Hf)_5_(Si,Al,B)_3_ (see text) [50]. The alloyed T2 is indicated in the maps in Figure 7 and Figure 8. The Π = (Nb,Ti)_5_Si_3_ is indicated in the maps in Figure 5, Figure 7 and Figure 8. The unalloyed Nb_5_Si_3_ in (**a**,**b**) corresponds to T1 in Figure 5, Figure 6, Figure 7, Figure 8, Figure 9, Figure 10 and Figure 11.

Creep data for unalloyed and alloyed tetragonal Nb_5_Si_3_ is shown in the Norton $\dot{\varepsilon }$ ∝ σ^n^ plots for 1473 K in Figure 12. The data sets (a) and (b) are for unalloyed αNb_5_Si_3_ prepared using (a) powder metallurgy processing (PM) and heat treatment (HT) with powders from crashed arc melted material and (b) arc melting + HT. The data shows that the creep rate increases as Nb is substituted by Cr, Hf and Ti and Si by Al and B. The positions of the unalloyed and alloyed silicides in Figure 12 are indicated in the VEC versus Δχ maps, for example see Figure 8. Increase in creep rate of Nb_5_Si_3_ results from alloying with Ti (compare T1 and Π) and with Cr, Hf and Ti and Al and B (compare T1 with T2 alloyed1 and T2 alloyed2) and these increases in creep rate are associated with decrease in the VEC and increase and decrease in the Δχ values (Figure 8).

## 3. Conclusions

This paper studied alloying behaviour and properties of Nb_5_Si_3_. The study used data for the silicide parameters VEC and Δχ and for the silicide solubility range, which was studied using the concentration X = Al + B + Ge + Si + Sn in (Nb,TM)_5_X_3_. Actual chemical compositions of tetragonal Nb_5_Si_3_ in developmental Nb-silicide based alloys, where in the silicide the Nb is substituted by Cr, Hf, Mo, Ta and Ti and the Si by Al, B, Ge and Sn individually or simultaneously, were used to calculate VEC, Δχ and X. Relationships between solvent and solute additions in Nb_5_Si_3_ and its parameters VEC and Δχ were found. Changes in the hardness and creep of tetragonal Nb_5_Si_3_ were related to the parameters VEC and Δχ. The conclusions of the research are as follows:

The concentration X was in the range 33.6 < X < 41.6 at.% and depended on the alloying addition(s). In Nb-24Ti-18Si-5Al-5Cr + 5Z alloys the single addition of element Z, where Z = Hf, Mo and Ta, or B, Ge and Sn, shifted the solubility range of X towards Nb (decreased X compared with the binary Nb_5_Si_3_) and Hf and Ge had the strongest effect. When B was in synergy with Hf or Mo or Sn the solubility exceeded 40.5 at.%. A shift towards higher X values was accompanied by a decrease of the values of the Δχ parameter of the Nb_5_Si_3_.

The Ge concentration in Nb_5_Si_3_ increased with its Ti concentration. The Hf concentration in Nb_5_Si_3_ increased and decreased with its Ti or Nb concentration respectively and its dependence on the latter was stronger. The B and Sn concentrations in the Nb_5_Si_3_ respectively decreased and increased with its Ti concentration and also depended on the concentrations of other alloying elements in the silicide.

The values of the parameters VEC and Δχ were in the ranges 4.11 < VEC < 4.45 and 0.103 < Δχ < 0.415. The parameter VEC described the alloying behaviour of Sn and the parameter Δχ described the alloying behaviour of B and Ge in Nb_5_Si_3_. The alloying behaviour of Nb_5_Si_3_ also was demonstrated in Δχ versus VEC maps.

Depending on alloying addition(s) the hardness of Nb_5_Si_3_ increased or decreased. Compared with the binary Nb_5_Si_3_, the hardness was increased when Ge was present in the silicide and decreased when Al, B and Sn were present in the silicide without Ge. The effect of Al depended on other elements substituting Si in the silicide. Sn reduced the hardness. The addition of Ti or Hf had a stronger negative effect on the hardness of Nb_5_Si_3_ than that of Cr in silicides without Ge. 

Deterioration of the creep of alloyed Nb_5_Si_3_ was linked with changes in the position of the Nb_5_Si_3_ in Δχ versus VEC maps. 

## Figures and Tables

**Table 1:** Experimental and calculated data for the CTE anisotropy [(CTE)_c_/(CTE)_a_] of binary and alloyed TM_5_Si_3_ silicides.

Silicide	Experimental	Calculated	Reference
Ti_5_Si_3_	4.39	-	[8]
	3.51	-	[7]
	3	-	[9]
	2.7	-	[10,11]
	1.68	-	[12]
W_5_Si_3_	3.3	-	[13]
Mo_5_Si_3_	2.21	-	[14]
	2	-	[15]
Zr_5_Si_3_	3.18	-	[10]
	2.79	-	[10]
	1.91	-	[12]
Ta_5_Si_3_	1.45	-	[7]
V_5_Si_3_	1.3	-	[16]
αNb_5_Si_3_	1.43	-	[8]
	1.25	-	[15]
	1.19	-	[17]
	-	1.12	[18]
	-	1.28	[19]
βNb_5_Si_3_	-	2.07	[18]
	-	1.52	[19]
α(Nb_50_Ti_12.5_)Si_37.5_	-	1.25	[19]
β(Nb_50_Ti_12.5_)Si_37.5_	-	1.64	[19]
(Ti,Zr)_5_Si_3_	1.22	-	[15]
(Mo,Nb)_5_Si_3_	1.25	-	[15]

**Table 2:** Solubility range of X (=Al + B + Ge + Si + Sn) in unalloyed and alloyed tetragonal Nb_5_Si_3_ in Nb-38Si binary and KZ5 type alloys * (see text and corresponding Δχ and VEC values of the silicide).

Alloy *	Solubility Range of X in Nb_5_Si_3_ (at.%)	Δχ		VEC	
		AC^+^	HT^+^	AC	HT
Nb-38Si	37.5–40.5	0.288	0.183	4.63	4.6
KZ5	35.3–36.4	0.363	0.322	4.41	4.42
KZ5 + 6Ta (=KZ6)	36.7–38.7	0.309	0.236	4.38	4.41
KZ5 + 5B (=TT4)	36.7–39.0	0.303	0.224	4.27	4.31
KZ5 + 6Ta + 5B (=TT5)	35.3–39.4	0.351	0.199	4.28	4.31
KZ5 + 5Hf (=JN1)	33.9–35.6	0.339	0.32	4.40	4.27
KZ5 + 5Ge (=ZF6)	33.6–34.3	0.415	0.39	4.38	4.42
KZ5 + 5Hf + 5Ge (=ZF9)	34.7–35.8	0.362	0.328	4.38	4.35
KZ5 + 5Hf + 5B (=TT7)	34.6–40.9	0.351	0.128	4.23	4.17
KZ5 + 5Sn (=ZX8)	34.7–36.9	0.38	0.302	4.37	4.45
KZ5 + 5Sn + 5Hf (=EZ8)	36.7–38.1	0.299	0.231	4.4	4.23
KZ5 + 5Sn + 5B (=TT6)	38.3–41.6	0.245	0.111	4.25	4.17
KZ5 + 5Sn + 5Ge (=OHS1)	35.5–38.8	0.351	0.23	4.39	4.31
KZ5 + 5Mo (=JG2)	34.1–36.2	0.410	0.330	4.40	4.33
KZ5 + 5Mo + 5B (=TT8)	37.3–41.4	0.289	0.103	4.36	4.11

AC^+^ = as cast, HT = heat treated. * Nominal alloy compositions (at.%): KZ5 = Nb-24Ti-18Si-5Al-Cr. ZF9 = Nb-24Ti-18Si-5Al-Cr-5Hf-5Ge; KZ6 = Nb-24Ti-18Si-5Al-Cr-6Ta. TT8 = Nb-24Ti-18Si-5Al-Cr-5Mo-5B; JN1 = Nb-24Ti-18Si-5Al-Cr-5Hf. TT6 = Nb-24Ti-18Si-5Al-Cr-5Sn-5B; ZF6 = Nb-24Ti-18Si-5Al-Cr-5Ge. TT7 = Nb-24Ti-18Si-5Al-Cr-5Hf-5B; TT4 = Nb-24Ti-18Si-5Al-Cr-5B. TT5 = Nb-24Ti-18Si-5Al-Cr-6Ta-5B; JG2 = Nb-24Ti-18Si-5Al-Cr-5Mo. OHS1= Nb-24Ti-18Si-5Al-Cr-5Ge-5Sn; ZX8 = Nb-24Ti-18Si-5Al-Cr-5Sn. EZ8 = Nb-24Ti-18Si-5Al-Cr-5Hf-5Sn.

**Table 3:** Calculated elastic moduli of TM_5_Si_3_ silicides and Nb_5_Si_3_ alloyed with Ti.

5-3 Silicide	E (GPa)	Reference
W_5_Si_3_	312	[52]
Mo_5_Si_3_	323	[52]
αTa_5_Si_3_	327.5	[52]
βTa_5_Si_3_	288.6	[52]
αNb_5_Si_3_	291	[42]
	314.3	[53]
	325	[54]
βNb_5_Si_3_	268.9	[42]
	276.9	[53]
Alloyed Nb_5_Si_3_	-	-
α(Nb_50_Ti_12.5_)Si_37.5_	313.8	[19]
β(Nb_50_Ti_12.5_)Si_37.5_	238.5	[19]

**Table 4:** Vickers hardness HV (kgf/mm^2^) of unalloyed Nb_5_Si_3_ and alloyed (Nb,Ti)_5_Si_3_ (Ti = 12.5 at.%) and unalloyed Ti_5_Si_3_. The HV *, HV^+^ and HV^C^ were calculated in GPa using HV* = (1 − 2ν)E/[6(1 + ν)], HV^+^ = 2[(G/B)^2^ G]^0.585^ − 3 and HV^C^ = 0.151 G [55] and the data for B, E, G and ν from [19,42] and were converted to Vickers hardness *.

5-3 Silicide	HV *	HV^+^	HV^C^	HV_measured_
αNb_5_Si_3_	2018	1558	1800	-
α(Nb,Ti)_5_Si_3_	2460	1964	1983	-
βNb_5_Si_3_	1708	1286	1640	1360
β(Nb,Ti)_5_Si_3_	1386	1023	1435	-
Ti_5_Si_3_	1590	1368	1365	1154

* To convert HV to GPa multiply by 0.009807.
